# Evaluation of the Hand and Wrist via Telephone and Video Visit

**DOI:** 10.7759/cureus.35322

**Published:** 2023-02-22

**Authors:** Rock P Vomer, Adam J Lewno, Imoh Udoh, Shirley Albano-Aluquin, George G. A Pujalte

**Affiliations:** 1 Department of Family and Community Health and Department of Orthopedics, Division of Sports Medicine, Duke University, Durham, USA; 2 Family Medicine, Mayo Clinic Jacksonville Campus, Jacksonville, USA; 3 Physical Medicine and Rehabilitation, University of Michigan, Ann Arbor, USA; 4 Department of Orthopedics, Division of Sports Medicine, Duke University, Durham, USA; 5 Department of Rheumatology, Penn State College of Medicine, Hershey, USA; 6 Departments of Family Medicine, and Orthopedics and Sports Medicine, Mayo Clinic, Jacksonville, USA

**Keywords:** access to health care, tele health, hand and wrist pain, wrist evaluation, hand evaluation

## Abstract

Background: The COVID-19 pandemic has highlighted the utility of telemedicine, with rapid incorporation throughout 2020. Telemedicine is a timely, safe, and effective means of evaluating, triaging, and treating patient conditions, including those of the musculoskeletal system. Hand and wrist complaints are frequently encountered in the primary care setting, and some can have serious consequences if not promptly diagnosed. Prior to the pandemic, over a quarter of the nation’s allopathic degree-granting medical schools had initiated telemedicine training as part of the preclinical phase of their curriculum, and about half had implemented it into clerkships prior to the pandemic. Despite rapid acceptance, increased ease of access, and prior attempts to incorporate telemedicine into the educational curriculum, telemedicine evaluation continues to pose challenges to both the patient and provider. This is likely due to a lack of established protocols outlining clinical data collection through a virtual interface. Although telemedicine requires the patient to perform a physical examination, it allows the physician to collect clinically important information while observing the patient in their home environment.

Aims: The aim of this paper is to provide a step-by-step method to evaluate and triage hand and wrist complaints.

Methods: Our group has created a step-by-step evaluation pathway to help physicians direct their patients through typical hand and wrist examination elements, including inspection, palpation, range of motion (ROM), strength, special, and functional testing.

Results: We have developed a table of evaluation questions and instructions and a glossary of images of each maneuver to facilitate hand and wrist examination via telemedicine.

Conclusion: This paper provides a guide for extracting clinically relevant information while performing telemedicine examinations of the hand.

## Introduction

In the setting of lockdowns and social distancing during a global pandemic, the practicality of telemedicine has become readily apparent with the rapid acceptance of its use to provide timely, safe, and effective care for patients. More importantly, telemedicine has been shown to be a viable mode for providing care for many patient complaints, including musculoskeletal conditions [1]. Despite this increased ease of access, a telemedicine evaluation poses challenges to both the patient and provider as nonverbal signs of pain and changes in function cannot be observed as they would during a traditional clinic visit. These subtle clues allow an experienced clinician to differentiate between common and rare pathology and quickly adjust the scope of their differential diagnosis to account for concerns beyond the chief complaint. While patient history can be collected over the telephone, the physical examination is performed by the patient [1]. Though their responses may lack details related to joint movement or tactile feedback from a special test, they are still highly valuable as they often focus the clinician on their perceived functional deficit. With a little creativity and collaboration with the patient, it is possible to conduct a thorough examination and provide effective care through telemedicine services.

Hand and wrist complaints are frequently encountered in the primary care setting, and some of these conditions may have serious consequences if not promptly diagnosed. Hand and wrist injuries commonly result from overuse or poor computer ergonomics, but may also be caused by traumatic injuries. Hand and wrist complaints have various causes that differ in adolescents and adults, and primary care physicians are likely to encounter all types.

Patient history allows the clinician to develop a differential diagnosis that is either supported or refuted by the results of the physical examination and diagnostic studies. Traditionally, a tailored physical examination to evaluate hand and wrist complaints involves inspection, palpation, range of motion (ROM) assessment, strength testing, sensation assessment, and special testing [2]. Telemedicine for hand and wrist examinations provides opportunities and benefits for both primary care providers and patients. For work-related ergonomics issues, video encounters provide a view into the patient's home workspace and computer setup, allowing the physician to provide tailored ergonomic adjustments. For traumatic injuries, telemedicine facilitates rapid triage and connection to the appropriate level of care. For patients, it allows for expanded access, reduces wait times for services, limits travel, and is cost-efficient. From a provider and health system perspective, it allows for efficient triage, monitoring, and improved allocation of medical resources.

As the use of telemedicine for patient encounters increases, protocols should be developed to provide direction on how to efficiently evaluate hand and wrist complaints via telephone and video. The American Medical Association has acknowledged the need for telemedicine training for medical students and residents [3]. The Liaison Committee on Medical Education’s Annual Medical School Questionnaire from 2015 to 2016 reported that more than one in four allopathic medical schools have added telemedicine education as part of the preclinical phase of their curriculum, and about half have implemented it into clerkship [4]. As medical schools continue to incorporate telemedicine training, standard protocols may be beneficial training resources. Additionally, protocols can be incorporated into continuing medical education for clinicians as virtual patient encounters become more prevalent. This paper outlines possible questions, responses, and video examination techniques to aid the clinician in conducting an effective hand and wrist examination during a telemedicine encounter.

## Materials and methods

**Figure 1:**
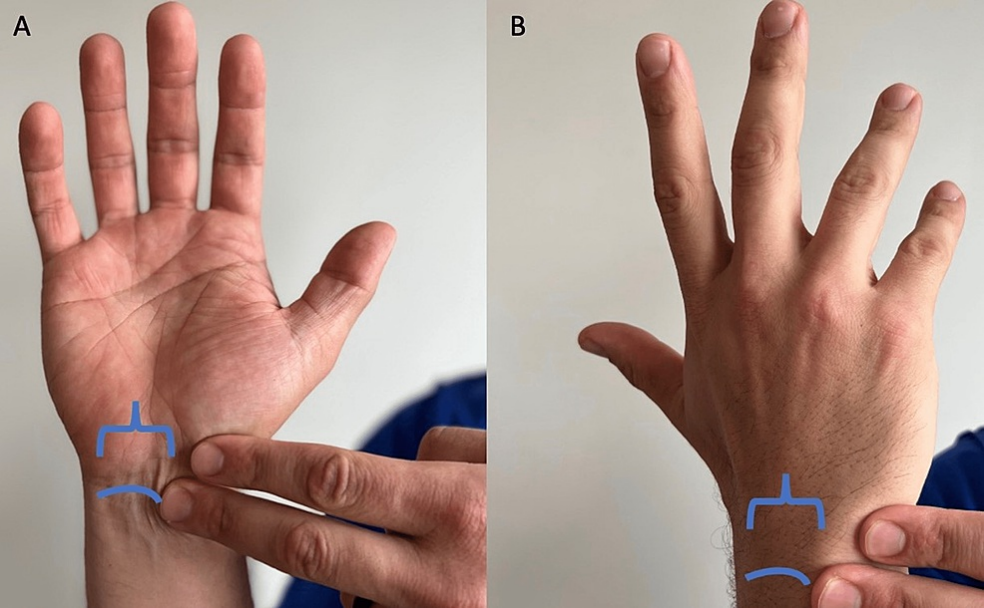
Anterior and posterior view of hand and wrist (A) Anterior view of the proximal row of carpal bones (curved line) and (B) distal row of carpal bones (bracket).

The patient should be instructed to sit facing the camera to provide the physician with an anterior view of the wrist and hand. First, inspect the palmar, dorsal, ulnar, and radial aspects of the hand and wrist (Figure 1).

**Figure 2:**
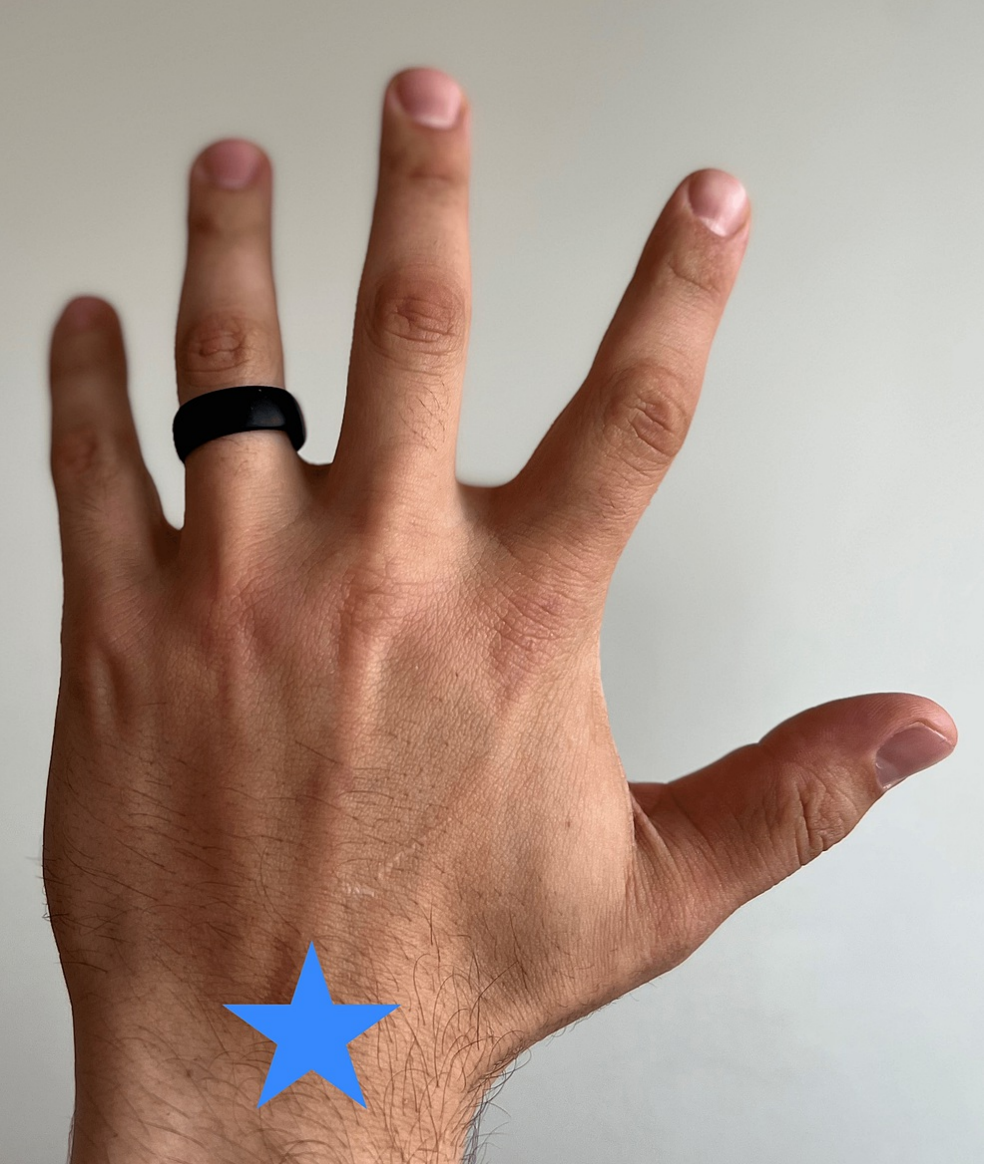
Observation for ganglion cyst A possible pathology of the dorsal wrist is a ganglion cyst; the possible location is demarcated by the star.

Look for asymmetry when comparing to the unaffected side to assess for malalignment, atrophy, swelling, ecchymosis, paleness, laceration, or venous distention. Closely observe the posterior aspect of the wrist for identifying a ganglion cyst (Figure 2).

**Figure 3:**
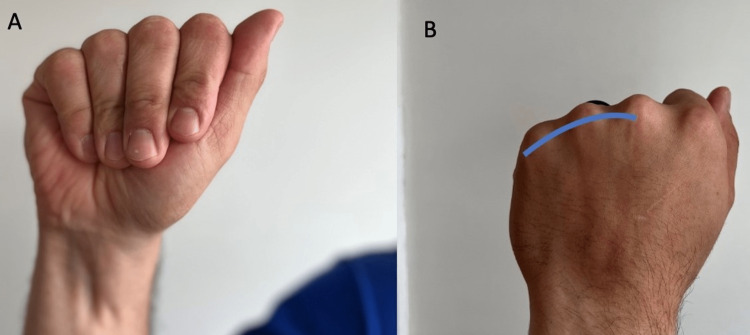
Anatomic alignment of the metacarpal bones (A) Normal anterior anatomic alignment, the digits point toward the base of the thumb. With a potential fracture or injury, the metacarpal or phalange could deviate from the normal alignment. (B) Normal posterior alignment of the metacarpal heads forms a smooth arc (curved line). If this is disrupted, a possible metacarpal fracture may be present.

Request the patient to make a fist and observe if the metacarpal-phalangeal joints form an arc (Figure 3). While their fist is still closed, observe if the finger pads can reach the palm and are in proper alignment in relation to the scaphoid. Have the patient show any concerning bumps or masses and compare these with those on the contralateral side.

**Figure 4:**
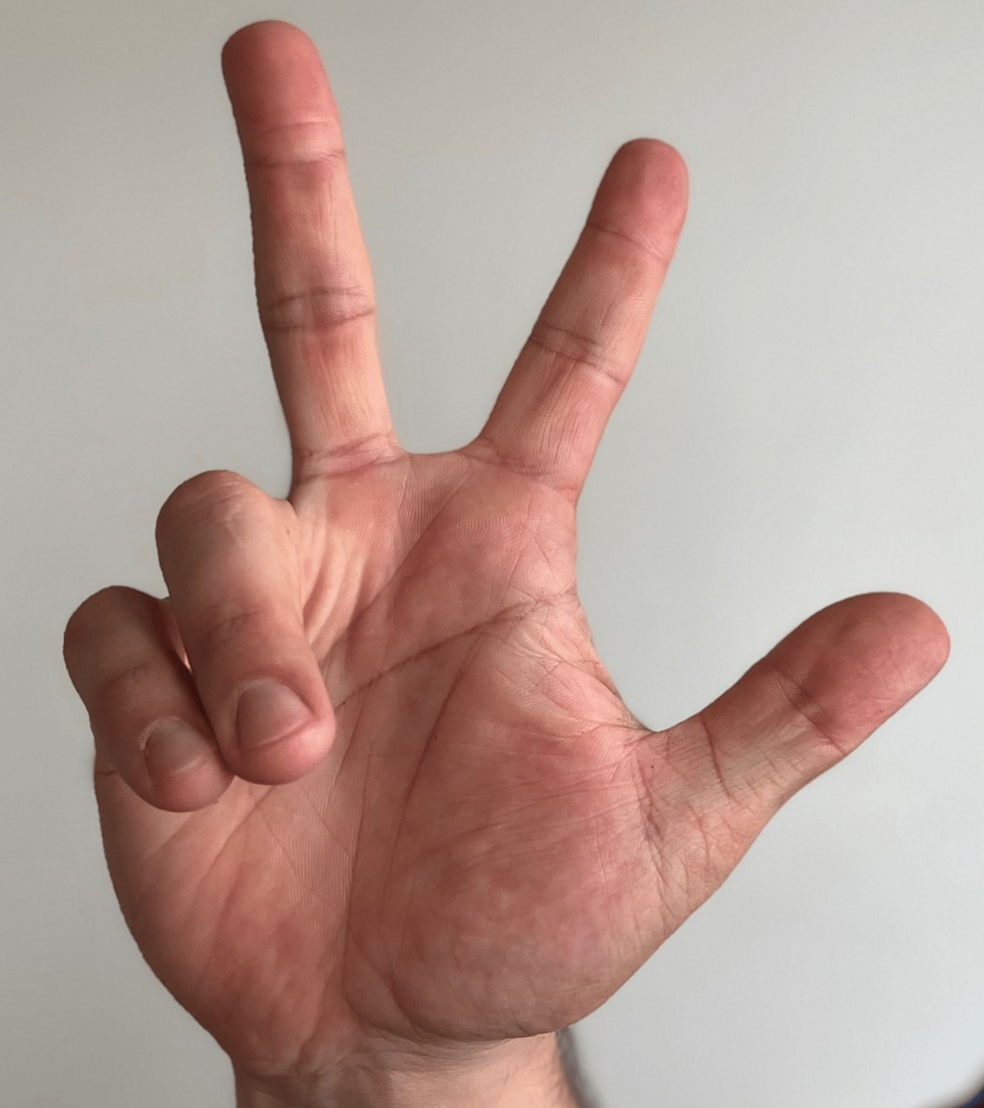
Ulnar claw This sign is suggestive of pathology of the ulnar nerve.

Pay close attention to the motion of the fingers while the patient opens and closes their hand when forming a fist. When the fist is closing, a lag or difficulty in closing the fist through the third digits may indicate an anterior interosseous nerve injury (benediction hand). Difficulty in the fourth and fifth digits upon opening the fist may suggest an ulnar nerve pathology (Figure 4).

Have the patient palpate the hand and wrist for areas of tenderness. Due to the close nature of the wrist structures, directing the patient may be difficult, and the location of the pain as indicated by the patient should be closely checked against anatomical structures in the region. The anatomical structures of interest include the carpal bones with directed attention to the scaphoid, snuff box, distal radial ulnar joint, radial styloid, ulnar styloid, first digit carpal metacarpal joint, extensor tendons with attention to extensor pollicis longus at listers tubercle and extensor carpi ulnaris (ECU), triangular fibrocartilage complex (TFCC) in the ulnar fovea, metacarpal bones, hook of the hamate, and the flexor carpi ulnaris (FCU) and radialis. 

Range of motion assessment should be performed first actively and then passively (if necessary), comparing affected and unaffected hands and wrists. It is important to report flexion (60°-90°), extension (40°-60°), ulnar deviation (30°-45°), radial deviation (15°-20°), supination (90°), and pronation (90°).

Strength testing to identify muscle weakness or pain should be performed as resisted movements. Resisted movements include wrist extension (extensor carpi radialis brevis/longus, ECU, extensor digitorum communis), ulnar deviation (extensor and flexor carpi ulnaris), radial deviation (extensor carpi radialis longus/radialis and flexor carpi radialis), wrist flexion (flexor carpi ulnaris and radialis), and pronation (pronator quadratus and proximal musculature).

**Figure 5:**
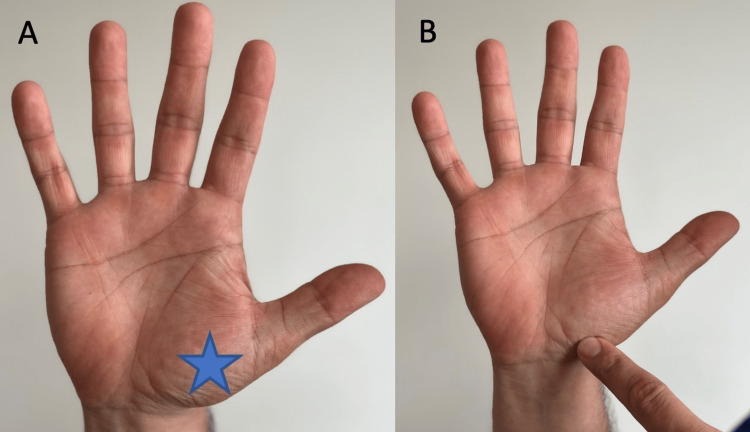
First carpometacarpal joint (A) Location of the first carpometacarpal joint; (B) if the patient appreciates squaring of the first carpal metacarpal joint it could be suggestive of osteophyte formation and osteoarthritis.

Radial wrist pathology is assessed by having the patient palpate over the radial styloid, extensor compartments, the distal-radial ulnar joint, the carpal bones with attention to the scaphoid, the carpometacarpal (CMC) joints, and the metacarpals. Sensor testing of the superficial radial nerve along the dorsum of the hand and the median nerve along the palmar aspect should be performed. Pain with thumb motion or grasping should prompt a CMC grind test, and pain would indicate potential CMC or scaphotrapeziotrapezoid (STT) osteoarthritis (Figure 5).

**Figure 6:**
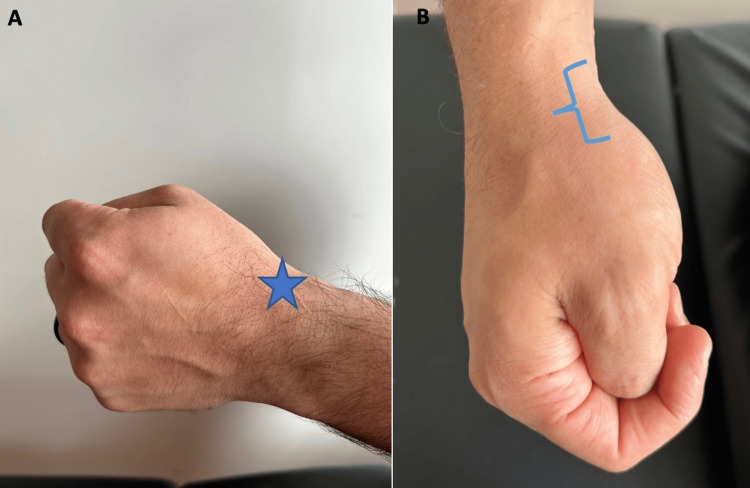
Finkelstein test (A) Lateral view of the Finkelstein test, pain along the first dorsal compartment of the wrist (star) is support of De Quervain tenosynovitis. (B) Superior view of the Finkelstein test, this position can be used to instruct the patient to palpate the anatomic snuff box (bracket).

Pain or weakness when the thumb is depressed in a fist that is ulnar deviated (Finkelstein test) may indicate first extensor compartment tenosynovitis or de Quervain tenosynovitis (Figure 6).

Pain with or without a clunking sensation when the scaphoid is compressed while the wrist moves from an ulnar to a radial position (modified Watson test) may indicate scapholunate instability. Care must be taken not to perform this test if there is any concern for a scaphoid fracture to prevent malalignment of fracture fragments. Weakness with or without pain during a handshake or grasp may indicate a carpal bone injury or carpal instability. Radial deviation of the first distal phalange while the metacarpophalangeal joint is stabilized would be concerning for an ulnar collateral ligament injury if there is abnormal gapping compared to the contralateral side.

**Figure 7:**
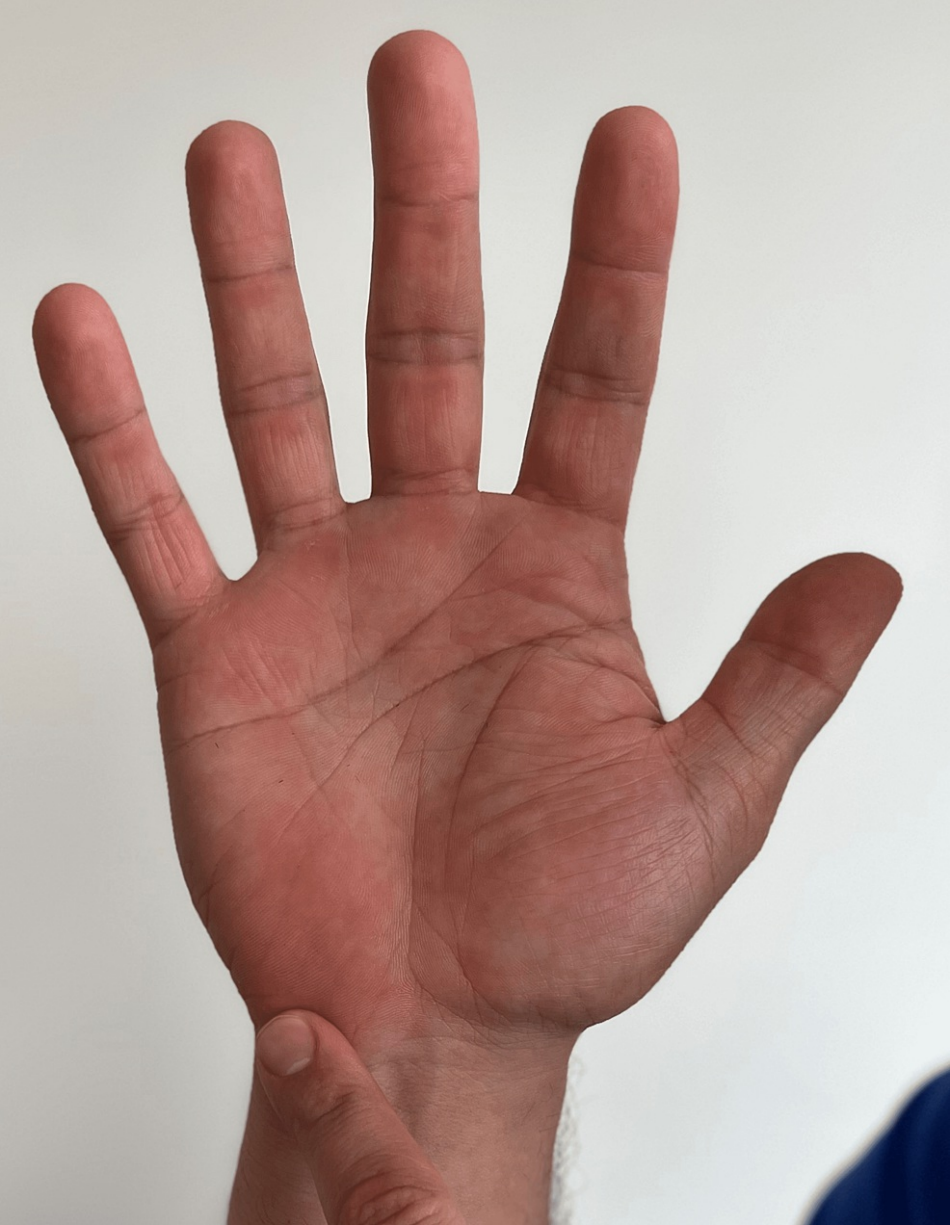
Guyon canal The Guyon canal is indicated by the finger point. Reproduction of symptoms at this area with palpation could indicate ulnar nerve entrapment, ulnar artery compromised or (hammer syndrome) or fracture of the hook of the hamate.

Ulnar wrist pathology is assessed by having the patient palpate over the ulnar styloid, ECU, ulnar fovea, and carpal bones. Pain on palpation and sustained compression at the Guyon canal are indicative of distal ulnar compression (Figure 7).

If there is a history of trauma to the ulnar aspect of the hand or repeated injury to the hook of the hamate, hammer syndrome should also be considered. Sensory testing along the fourth and fifth palmar digits should be performed. Ulnar deviation causing pain within the wrist crease may indicate a complex triangular fibrocartilage injury. Pain on palpation in the ulnar fovea, with or without ulnar deviation, and ulnar wrist pain during a push may further indicate a triangular fibrocartilage complex injury. Pain or weakness localized to the ulnar aspect of the wrist crease during supination or pronation may indicate ECU tendinosis. If a popping or sliding sensation is experienced during this maneuver, ECU subluxation may also be occurring, which is often accompanied by soft tissue swelling.

**Figure 8:**
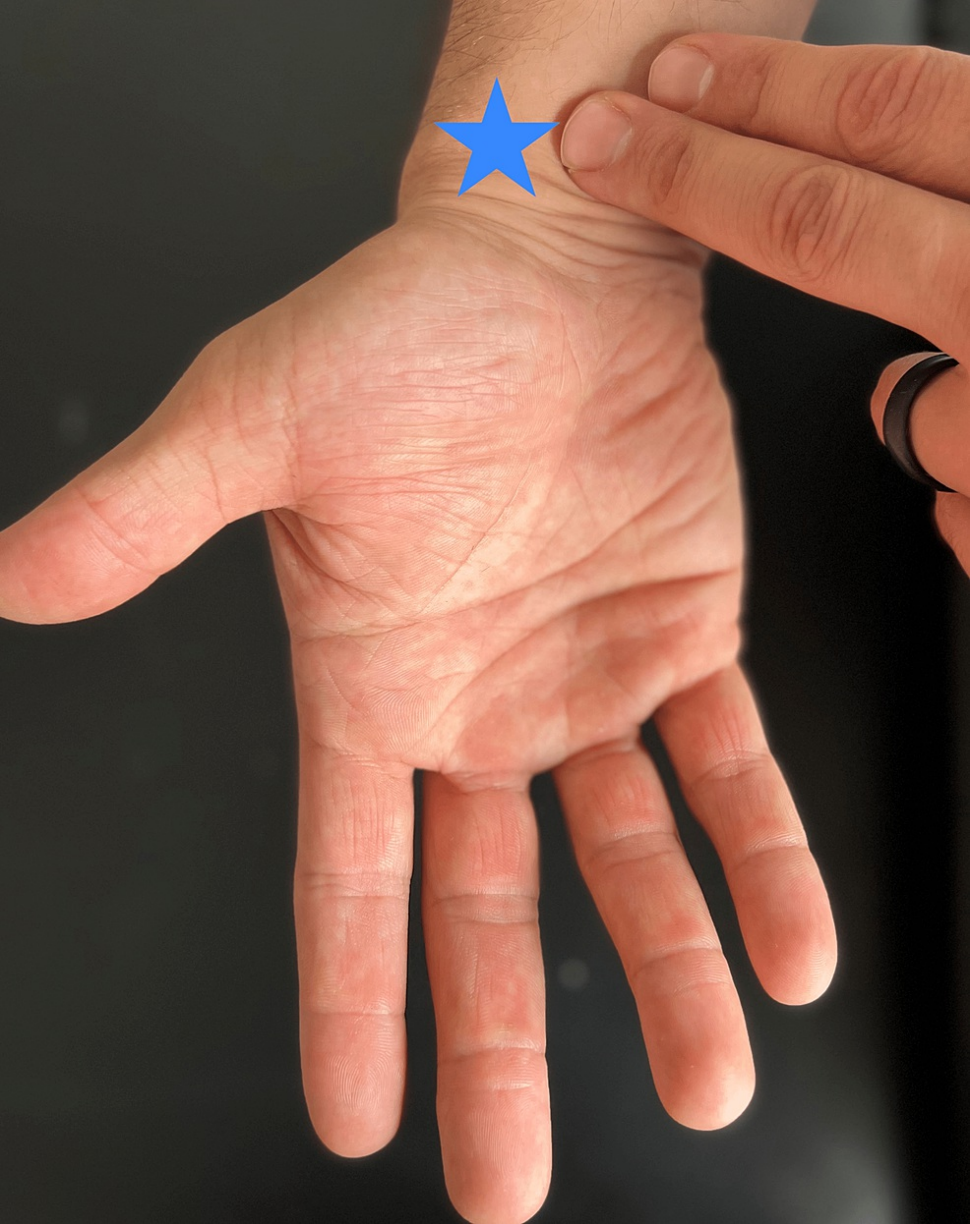
Tinel's sign Percussion at the transverse carpal ligament (star) that reproduces radicular symptoms into the hand suggest median nerve involvement.

**Figure 9:**
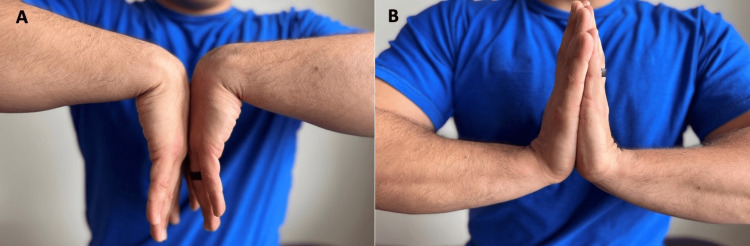
Phalen and reverse Phalen tests (A) Phalen test; (B) reverse Phalen test. These tests are positive when they reproduce radicular symptoms in the median nerve distribution in the hand.

Additional areas of neuromuscular pathology at the wrist can be detected with the careful guidance of palpation. Pain or paresthesias with palmar wrist crease compression (the Durkin test) or tapping of this region (the Tinel sign at the wrist) would indicate potential carpal tunnel syndrome (Figure 8). A similar sensation while pressing both wrists together with fingertips pointing to the floor (Phalen test) further supports concern for carpal tunnel syndrome (Figure 9).

**Figure 10:**
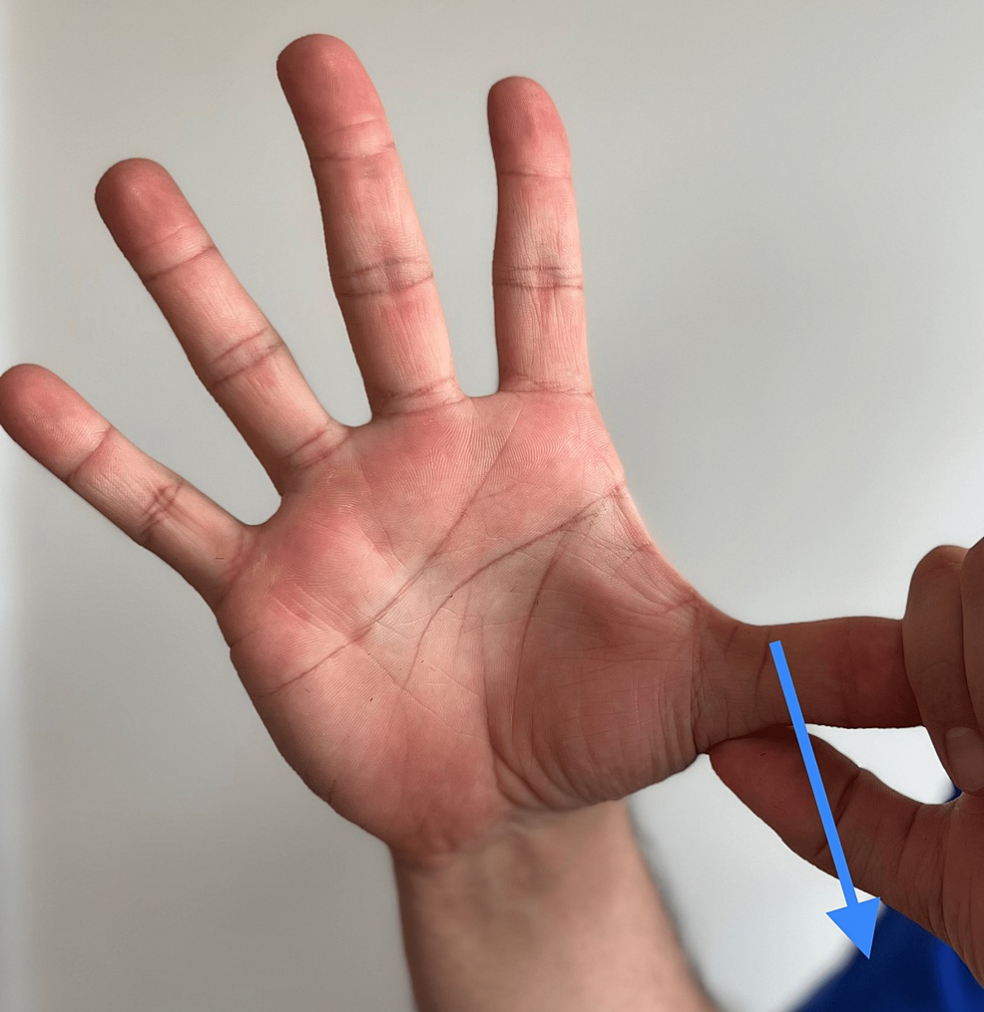
Valgus stress test of the thumb Valgus stress test of the ulnar collateral ligament of the thumb can be performed by instructing the patient to gap the first carpal metacarpal joint (arrow indicates the motion vector). A positive test is an increase in joint gapping and pain reproduction.

If the patient is unable to form an okay sign, an anterior interosseous nerve injury should be suspected. Pain or paresthesias experienced along the dorsum of the wrist when a watch band is tightened may indicate superficial radial nerve compression. If a watch is not available, the patient can firmly wrap their other hand around their wrist to mimic a watch band with similar results. If the patient reports ulnar-sided thumb pain after a fall, testing the integrity of the ulnar collateral ligament of the thumb is indicated. This can be accomplished with valgus stress testing (Figure 10).

## Results

**Figure 11:**
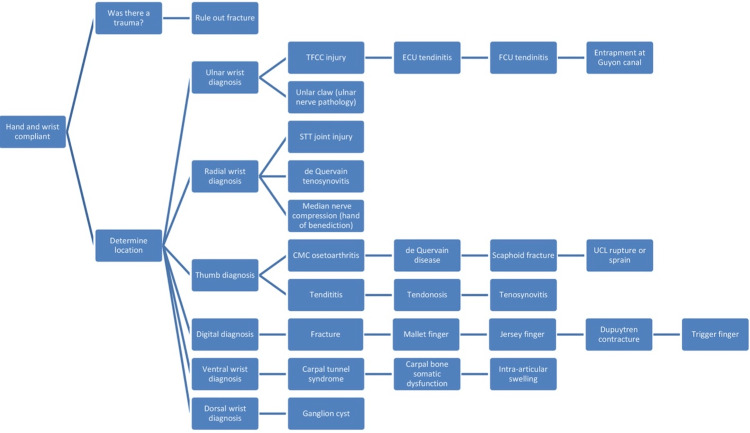
Differential diagnosis flow chart Abbreviations for Important Anatomical Structures of the Hand and Wrist. CMC: carpometacarpal; ECU: extensor carpi ulnaris; FCU: flexor carpi ulnaris; STT: scaphotrapeziotrapezoid; TFCC: triangular fibrocartilage complex; UCL: ulnar collateral ligament.

Our group has created a step-by-step evaluation pathway to help physicians direct their patients through typical hand and wrist examination elements, including inspection, palpation, range of motion, strength, and special and functional testing. We have also created a differential diagnosis flow chart to aid the clinician in the evaluation of hand-wrist complaints (Figure 11). We have developed a table of evaluation questions and instructions and a glossary of images of each maneuver to facilitate hand and wrist examination via telemedicine. Table 1 outlines questions and instructions that can be used during a telephone encounter to evaluate the hand and wrist and the possible implications of the patient’s responses [5-18]. Figures 2-11 demonstrate maneuvers described in Table 1.

**Table 1:** Hand and wrist evaluation by telephone Abbreviations: CMC, carpometacarpal; ECU, extensor carpi ulnaris; FCU, flexor carpi ulnaris; ROM, range of motion; STT, scaphotrapeziotrapezoid; TFCC, triangular fibrocartilage complex; UCL, ulnar collateral ligament.

What to ask	Possible responses	Implications of responses
Inspection		
“Do you see any differences between your left and right wrist?” (Figure 1)	Affirmative response	Possible carpal bone, distal radial ulnar joint, ganglion cyst, carpal boss, or normal variant.
“Do you see any sunken, swollen, bruised, red, or pale areas on your wrist?”	Affirmative response	Sunken areas, possible atrophy; swollen, bruised, or red areas, possible injury or infection; pale areas, possible arterial supply limitations.
“Do you see any band-like structures in your palm?”	Affirmative response	Possible Dupuytren contracture [16].
“Do you see any finger deformity?”	Affirmative response	Possible fracture, mallet finger, or jersey finger [6].
“Do you have a compressible bump along your wrist? (Figure 2)	Affirmative response	Possible synovial ganglion cyst or intra-articular swelling.
“When you make a fist, do all your fingers point to the base of your thumb?” (Figure 3)	Negative response	Potential metacarpal or phalange injury.
“When you make a fist, do your first knuckles form a slight arch?” (Figure 3)	Negative response	Possible metacarpal fracture or sagittal band injury [15].
ROM assessment		
“Can you make a make a fist with all your fingers touching your palm?”	Negative response	Possible fracture along the phalanges, degenerative joint disease, or infection.
“Can you extend your wrist so your fingers point to the ceiling and flex your wrist so your fingers point to the floor?”	Negative response	Followed up with review of dorsal wrist for extension limitation or palmar/volar wrist for flexion wrist; possible osteoarthritis, somatic dysfunction of carpal bones, synovitis, ganglion cyst, trauma, or tendinopathy.
“Can you turn your wrist so your fingers point toward and away from your body?”	Negative response	Followed up with review of parallelogram effect of radius and ulna; possible osteoarthritis, somatic dysfunction of carpal bones, synovitis, ganglion cyst, trauma, or tendinopathy
“Does the nonaffected wrist have more flexion, extension, or turning motion than the affected wrist?”	Affirmative response	Note how ROM loss effects function and if affected wrist is on the dominant hand; possible osteoarthritis, somatic dysfunction of carpal bones, synovitis, ganglion cyst, trauma, or tendinopathy [18].
“Do you have any joint squaring at the base of your thumb?” (Figure 5)	Affirmative response	Instruct the patient to perform grind test of the CMC; pain indicates possible osteophyte formation or CMC osteoarthritis [14].
“Did you experience acute pain with weakness of wrist or finger extension?	Affirmative response	Look for ecchymosis or visible mass; possible extensor tendon injury.
Palpation		
“Does it hurt to push along the base of your thumb?” (Figure 5)	Affirmative response	Possible CMC, scaphoid, trapezium, trapezoid, or STT joint injury, distal intersection, de Quervain disease, tenosynovitis, tendinitis, tendinosis, or fracture; if history of trauma, possible scaphoid fracture [5,11].
“Does it hurt to press the soft spot just before the base of your thumb?”	Affirmative response	Most likely scaphoid injury, but possible CMC joint or first extensor compartment pathology [11].
“Does it hurt if you compress and grind your thumb in a circular motion?”	Affirmative response	Positive grind test; possible osteoarthritis or scaphoid injury or fracture [11,14].
“Does it hurt the thumb side of your wrist when you make a fist with your thumb inside the fist and tilt your fist so your pinkie points to your elbow?” (Figure 6)	Affirmative response	Positive modified Finkelstein test; possible first extensor compartment tenosynovitis (de Quervain disease) or CMC joint pathology [5].
“Does it hurt when you bend the thumb knuckle closest to the first webspace away from your hand? Is there swelling or bruising along the pinkie side of your thumb near this location?” (Figure 10)	Affirmative response	Possible UCL injury with increased concern if there is ecchymosis or a history of trauma; without trauma, possible osteoarthritic or rheumatologic pathology [13].
“What tasks have you found difficult due to weakness, pain, or ROM limitation of the wrist” For strength testing, the clinician may also ask patient to reproduce tasks/movements/exercises over the phone and describe weakness or pain felt.	Response may indicate strength problems in the elbow (e.g., “I am unable to lift or swing my golf club due to pain on the thumb side of my wrist)”	Depending on the description of tasks in which the patients is having weakness/pain, some pathologies are first extensor compartment tenosynovitis, CMC joint pathology.
Strength testing		
“With your palm facing the ceiling, does it hurt when you press along the pinkie side of your wrist?”	Affirmative response	Possible TFCC, ECU, ulnar nerve, or FCU pathology; have the patient perform piano key test to assess for TFCC tears [7].
“With your palm facing the ceiling, is there pain when you press in the soft spot on the outside of the pinkie side of your wrist or when you tilt your wrist sideways as if trying to point to your belly button?”	Affirmative response	Most likely TFCC injury, but possible carpal pathology.
“Do you have pain on the pinkie side of your wrist when you do a push up or push something away? (Figure 7)	Affirmative response	Possible TFCC injury or ulnar nerve pathology within the Guyon canal [9].
“Do you have pain, swelling, or a snapping sensation when you turn your palm from face up to face down or the reverse?”	Affirmative response	Possible ECU injury or subluxation.
“With your palm facing the ceiling, does it hurt or cause a tingling in your fingers when you press the wrist crease below your pinkie?	Affirmative response	Possible ulnar nerve pathology within the Guyon canal; less likely, ulnar vasculature pathology (hammer syndrome).
“What tasks have you found difficult due to weakness, pain, or ROM limitation of the wrist?” For strength testing, the clinician may also ask patient to reproduce tasks/movements/exercises over the phone and describe weakness or pain.	Response may indicate strength problems in the elbow (e.g., “I am unable to grip the handlebars of my bike because my hand feels weak and my fingers are tingling.”)	Depending on the description of tasks in which the patient is having weakness or pain, possible ulnar nerve compression or TFCC or ECU tendinosis or subluxation.
Special and functional testing		
“When you make a fist, is there a lag or difficulty in closing the 1^st^-3^rd^ fingers?”	Affirmative response	Possible anterior interosseous nerve (benediction hand) [10].
“When you make a fist, is there difficulty in closing the 4^th^ and 5^th^ fingers?” (Figure 4)	Affirmative response	Possible ulnar nerve pathology (ulnar claw).
“Did you feel a pop or rip in your finger at the time of injury? Are you unable to bend the last joint of your finger?”	Affirmative response	Possible jersey finger injury or avulsion of the flexor digitorum profundus.
“Did your pain begin after a fall?”	Affirmative response	Possible fracture or injury based on the nature of fall and noted deformities.
“When you open your hand, does one finger get stuck?”	Affirmative response	Possible trigger finger.
“When your palm is facing the ceiling, does it hurt or cause tingling in your fingers when you press on the wrist crease?”	Affirmative response	Possible carpal tunnel syndrome; if pain or tingling is on the ulnar side, possible ulnar compression.
“With your palm is facing the ceiling, tap your wrist crease. Do your fingers tingle?” (Figure 8)	Affirmative response	Positive Tinel sign; possible carpal tunnel syndrome [17].
“When you press the top of your hands together so your fingers point towards the ground, do your fingers tingle?” (Figure 9)	Affirmative response	Positive Phalen maneuver; possible carpal tunnel syndrome [17].
“If you clench your fist and have another person compress the arteries on either side of your wrist, is there a delay in the return of color in your hand?”	Affirmative response	Positive Allen test; possible arterial disruption in the deep and superficial arterial arches in the hand [12].
“Do you experience pain when you bend your thumb away from your index finger?”	Affirmative response	Possible UCL rupture or sprain.
“If you put on a watch or wrap your hand around your wrist, do you feel tingling or pain along the top of your hand?”	Affirmative response	Possible superficial radial nerve compression; less likely, injury to the 1^st^ or 2^nd^ extensor tendon compartments.
“Do you feel burning or tingling in your fingers and arm?”	Affirmative response	At 1^st^ palmar interossei, probable median nerve compression; at 4^th^ and 5^th^ palmar interossei, probable ulnar pathology, though can include the ulnar dorsal hand/wrist; at dorsal wrist and hand, probable radial nerve compression; have the patient perform a Spurling test to rule out cervical radicular pain[8].

## Discussion

Musculoskeletal diagnoses are important for patients with hand and wrist pain. The ability to evaluate hand and wrist injuries from a distance can improve patient access to timely and effective care. When imaging is needed, there are specific findings that can be described via telephone or seen on video, such as decreased range of motion or marked swelling, that can help clinicians know when and which tests or imaging modalities to order. The patient’s affected hand and wrist should always be compared with the unaffected side, with close inspection by video if possible. Telephone and video visits allow for efficient triaging, decreasing the need for more expensive procedures, imaging, or referrals. Evaluation, assessment, and treatment of hand and wrist injuries are possible via telemedicine with patient collaboration.

Telehealth is becoming rapidly accepted as a method for patients to connect with their physicians remotely and across the limitations posed by a pandemic. Clinicians should learn how to utilize this resource. As billing and coding for telehealth improve, there will be a shift towards an increased volume of this visit type. While there are limitations to telehealth encounters, they are surmountable, and it is undeniable that hand and wrist complaints can be addressed through such visits. Our guide serves as an easy-to-follow, step-by-step guide to help physicians obtain the necessary information to triage and treat conditions of the hand and wrist.

The main limitations of telehealth are that telephone encounters are limited to only patient-reported histories, and physicians will need to improve their history-taking skills. Video visits have the added visual component of a traditional visit but lack the ability to palpate the patient. During these encounters, physicians would need to carefully instruct the patients on physical examination maneuvers. Patients who would have difficulty accessing in-person visits and would require promoted triage or who would prefer increased access to their physician are likely to use telehealth encounters for the evaluation and management of hand and wrist complaints. In the future, we will be conducting validation studies to evaluate the accuracy of telehealth encounters on the hand and wrist.

## Conclusions

The COVID-19 pandemic has demonstrated the utility of having creative modes of healthcare delivery, through avenues such as telemedicine, for patients with musculoskeletal conditions. In the primary care setting, clinicians frequently encounter patients with hand and wrist complaints. Telemedicine can offer providers a unique opportunity to extract clinically relevant information through a virtual interface to diagnose and treat patients with hand or wrist pathology. This process becomes more effective when providers can direct patients through the steps of a virtual encounter by following an established protocol, such as the step-by-step hand and wrist evaluation pathway described in this paper. Efforts to provide trainees at the medical student and resident levels with the skills to accurately connect and examine patients with hand and wrist complaints remotely will prove advantageous in an era where the practicality of telehealth encounters is becoming more commonly recognized.
